# Caspase‐1 inflammasome activity in patients with *Staphylococcus aureus* bacteremia

**DOI:** 10.1111/1348-0421.12738

**Published:** 2019-10-16

**Authors:** Gunlög Rasmussen, Berhane Asfaw Idosa, Anders Bäckman, Stefan Monecke, Kristoffer Strålin, Eva Särndahl, Bo Söderquist

**Affiliations:** ^1^ Department of Infectious Diseases Örebro University Hospital Örebro Sweden; ^2^ School of Medical Sciences, Faculty of Medicine and Health Örebro University Örebro Sweden; ^3^ iRiSC ‐ Inflammatory Response and Infection Susceptibility Centre, Faculty of Medicine and Health Örebro University Örebro Sweden; ^4^ Department of Clinical Research Laboratory, Faculty of Medicine and Health Örebro University Sweden; ^5^ Leibniz Institute of Photonic Technology (IPHT) InfectoGnostics Research Campus Jena Jena Germany; ^6^ Department of Infectious Diseases Karolinska University Hospital Stockholm Sweden; ^7^ Department of Medicine Huddinge Karolinska Institutet Stockholm Sweden

**Keywords:** caspase‐1, NLRP3, sepsis, *Staphylococcus aureus*

## Abstract

The inflammasome is a multiprotein complex that mediates caspase‐1 activation with subsequent maturation of the proinflammatory cytokines IL‐1β and IL‐18. The NLRP3 inflammasome is known to be activated by *Staphylococcus aureus*, one of the leading causes of bacteremia worldwide. Inflammasome activation and regulation in response to bacterial infection have been found to be of importance for a balanced host immune response. However, inflammasome signaling *in vivo* in humans initiated by *S. aureus* is currently sparsely studied. This study therefore aimed to investigate NLRP3 inflammasome activity in 20 patients with *S. aureus* bacteremia (SAB), by repeated measurement during the first week of bacteremia, compared with controls. Caspase‐1 activity was measured in monocytes and neutrophils by flow cytometry detecting FLICA (fluorescent‐labeled inhibitor of caspase‐1), while IL‐1β and IL‐18 was measured by Luminex and ELISA, respectively. As a measure of inflammasome priming, messenger RNA (mRNA) expression of *NLRP3*, *CASP1* (*procaspase‐1*), and *IL1B* (*pro‐IL‐1β*) was analyzed by quantitative PCR. We found induced caspase‐1 activity in innate immune cells with subsequent release of IL‐18 in patients during the acute phase of bacteremia, indicating activation of the inflammasome. There was substantial interindividual variation in caspase‐1 activity between patients with SAB. We also found an altered inflammasome priming with low mRNA levels of *NLRP3* accompanied by elevated mRNA levels of *IL1B*. This increased knowledge of the individual host immune response in SAB could provide support in the effort to optimize management and treatment of each individual patient.

**Abbreviations**
CC: clonal complex
DAMP: damage‐associated molecular pattern
FLICA: fluorescent‐labeled inhibitor of caspase‐1
IQR: interquartile range
MFI: mean fluorescence intensity
NLR: NOD‐like receptor
PAMP: pathogen‐associated molecular pattern
PRR: pattern‐recognition receptor
SAB: *Staphylococcus aureus* bacteremia
SOFA: Sequential Organ Failure Assessment
TLR: Toll‐like receptor

## 1 INTRODUCTION

Host innate immune response plays a crucial role in defending the organism against infection. Pattern‐recognition receptors (PRRs), such as Toll‐like receptors (TLRs) and NOD‐like receptors (NLRs), are able to recognize a wide range of microbial components, known as pathogen‐associated molecular patterns (PAMPs), as well as sterile molecules of stress and danger, so‐called damage‐associated molecular patterns (DAMPs). PRRs thus act as a first line of defense against infections and host damage. While TLRs are membrane‐bound, NLRs act as intracellular sensors for pathogens and stress.^1^, ^2^ Upon sensing, some NLRs oligomerize and assemble with the adaptor protein ASC (apoptosis‐associated speck‐like protein containing a CARD) and procaspase‐1 to form multiprotein complexes known as inflammasomes. The assembly mediates caspase‐1 activation, which subsequently cleaves the proinflammatory cytokines IL‐1β and IL‐18 into their active forms.^3^, ^4^, ^5^

The NLRP3 inflammasome is one of the best characterized inflammasomes. It is activated by a variety of different pathogens,^6^, ^7^, ^8^, ^9^ but also by danger molecules, such as adenosine triphosphate, uric acid, asbestos, and silica.^4^, ^7^, ^10^, ^11^ In contrast to most other PRRs activated by direct interaction with a ligand, the NLRP3 inflammasome seems to act on common sensors of cellular damage, such as potassium efflux, reactive oxygen species, lysosomal disruption, and calcium signaling,^5^, ^11^, ^12^ signals which are secondary to infection or cellular stress. In general, activation of the NLRP3 inflammasome is a two‐step process. The first priming signal is a nuclear factor‐kappa B‐mediated transcription of NLRP3 protein and pro‐IL‐1β induced via, for example, TLR signaling. A second NLRP3‐sensing signal is then required to activate the NLRP3 protein, leading to inflammasome assembling.^5^, ^12^, ^13^

*Staphylococcus aureus* is a major pathogen and one of the leading causes of bacteremia worldwide.^14^ It is also one of the pathogens known to activate the NLRP3 inflammasome.^6^, ^7^ The pathogenicity of *S. aureus* is complex and involves a large repertoire of virulence factors, such as adhesion proteins, secreted toxins and enzymes, and regulatory factors.^15^ Adhesion proteins may be of importance for invasiveness,^15^ whereas pore‐forming toxins, mainly hemolysins and leukotoxins, have been found to activate the NLRP3 inflammasome and elicit an inflammatory response.^6^, ^16^, ^17^, ^18^, ^19^ Besides the virulence factors displayed by the pathogens, host‐related factors play a crucial role in disease manifestation and recovery.

Complications of *S. aureus* bacteremia (SAB) are common, and include infective endocarditis, septic arthritis, osteomyelitis, and local extension of infection, such as deep‐seated abscesses.^20^ It is important to identify these complications, as a complicated disease course will strongly affect patient management regarding antibiotic treatment and other therapeutic strategies.^21^ In severe cases, bacteremia can also lead to a life‐threatening organ dysfunction caused by a dysregulated host response to the infection (i.e. sepsis)^22^ that is characterized by both proinflammatory and anti‐inflammatory responses occurring early and simultaneously.^23^ The discovery of PRR signaling through DAMPs and PAMPs has improved the understanding of sepsis pathophysiology mechanisms.^24^ Overall, inflammasome activation as well as its regulation in response to bacterial infection is necessary for a balanced host response.^25^, ^26^ Inflammasome activation initiated by *S. aureus* has so far mainly been studied in murine models and *in vitro* in monocytes, macrophages, and their corresponding cell lines.^6^, ^16^, ^17^, ^18^, ^19^ Inflammasome activation in neutrophils is less studied but highly relevant, because neutrophils account for the majority of leucocytes and are recruited early to the site of infection. Furthermore, knowledge on inflammasome signaling *in vivo* in humans is currently sparse.

The aim of this study was therefore to elucidate the NLRP3 inflammasome activity in patients with SAB at both transcription and effector levels during the acute phase of SAB, by investigating caspase‐1 activity in neutrophils and monocytes, and the production of its downstream cytokines IL‐1β and IL‐18. Messenger RNA (mRNA) expression of *NLRP3*, *CASP1 (procaspase‐1)*, and *IL1B (pro‐IL‐1β)* was analyzed, as a measure of inflammasome priming.

## 2 MATERIALS AND METHODS

Patients with SAB were prospectively included from July 2012 to June 2014 at Örebro University Hospital, Sweden, when at least one of the four blood culture bottles routinely collected on hospital admission yielded growth of *S. aureus*. Blood cultures were incubated using the BACTEC system (Becton Dickinson, Franklin Lakes, NJ), and species identification was performed by matrix‐assisted laser desorption ionization‐time of flight mass spectrometry (Microflex LT and Biotyper 3.1; Bruker Daltonics, Bremen, Germany). Susceptibility testing was performed using the disc diffusion method according to the 2014 EUCAST guidelines.

From enrolment on day 1 (the day blood culture signaled positive), blood sampling was performed on five occasions: days 1, 2, 3, 5, and 7, with a clinical assessment carried out simultaneously. Exclusion criteria were age <18 years, HIV infection, neutrophils <1 × 10^9^/L, and immunosuppressive treatment (i.e. chemotherapeutics other than methotrexate, corticosteroids equivalent to ≥10 mg prednisolone, or tumor necrosis factor‐α inhibitors).

Sepsis was defined as an acute change in Sequential Organ Failure Assessment (SOFA) score of ≥2 points due to the infection, according to the Sepsis‐3 criteria.^22^ SOFA score was calculated from the worst recorded values within 48 hr of hospital admission. Patients with SAB were also categorized as having noncomplicated or complicated bacteremia. Complicated SAB was defined as either the presence of metastatic infection (e.g. infective endocarditis or osteomyelitis), extension of infection beyond the primary focus (such as an abscess), embolic stroke, attributable mortality (i.e. hospital‐related death within 60 days of admission in a patient with persistent signs or symptoms of infection), or recurrent SAB within 12 weeks.^20^ All other cases were defined as uncomplicated SAB. In total, 26 healthy blood donors between the ages of 18 and 60 were included as controls, each sampled once.

### 2.1 Caspase‐1 detection

Peripheral blood from patients with SAB and controls (blood donors) was collected in VACUETTE EDTA tubes and stained for caspase‐1 activity with FAM‐YVAD‐FMK (fluorescent‐labeled inhibitor of caspase‐1 [FLICA]; Immunochemistry Technologies, Bloomington, MN) for 1 hr at 37°C, as previously described.^8^ Leukocytes were labeled with RPE‐CY5‐conjugated mouse antihuman CD45 (DakoCytomation, Glostrup, Denmark), RPE‐conjugated mouse antihuman CD11b (DakoCytomation), and ECD‐conjugated mouse antihuman CD14 (Beckman Coulter; Immunotech, Marseille, France) to differentiate between the various leukocyte populations. Neutrophils and monocytes were separated based on side scatter, CD45, and CD14 gating. Furthermore, the data were confirmed based on side scatter, CD11b, and CD14 gating. Nonspecific binding was analyzed using an isotypic control IgG1 FITC/RPE/RPE‐CY5 (DakoCytomation), and found to be nonsignificant. Caspase‐1 activity was determined via flow cytometry (FC 500 Beckman Coulter, Fullerton, CA) by detecting FLICA fluorescence as mean fluorescence intensity (MFI) value for each sample. Acquisition of data was set to count a total of 50,000 events, and the Kaluza software package was used to analyze the data.

### 2.2 Measurement of IL‐18 and IL‐1β

Whole blood from SAB patients and controls was collected in EDTA tubes and centrifuged at 2000***g*** for 10 min to collect plasma for the detection of cytokine levels. Plasma was stored at −80°C pending analysis. Levels of IL‐1β were measured by a commercially available IL‐1β MILLIPLEX human Cytokine kit according to manufacturer's instructions (Millipore Corporation, Billerica, MA), and analyzed with the Luminex 200 system (MAP technology, Austin, TX). An ELISA kit (Medical & Biological Laboratory Co Ltd, Nagoya, Japan) was used to measure plasma concentration of IL‐18 according to manufacturer's instructions. The optical density was measured on a Multiskan Ascent plate reader (Thermo Labsystems, Norwich, UK) at 450 nm.

### 2.3 RNA extraction, reverse transcription, and quantitative PCR

The mRNA expressions of *NLRP3*, *CASP1*, and *IL1B* were studied using venous blood collected directly in PAXgene Blood RNA tubes (Preparalytic GmbH, Qiagen Group, Hilden, Germany). The tubes were stored after sampling at −80°C pending further analysis. RNA isolation and complementary DNA (cDNA) preparation were performed as previously described according to manufacturer's instruction. Briefly, 100 ng of purified (Qiagen, PreAnalytiX) total RNA was transcribed to cDNA (Applied Biosystems/Life Technologies) prior to quantitative PCR. The cDNA (2 µl) was determined in the following TaqMan gene expression assays (FAM‐labeled MGB probes; Applied Biosystems/Life Technologies Europe BV, Stockholm Sweden): *NLRP3* (Hs00918082_m1, Lot:1235590), *CASP1* (Hs00354836_m1, Lot:1243286), and *IL1B* (Hs01555410_m1, Lot:1240086). The samples were run in duplicates (20 µl) in a 96 well fast‐format on an ABI7900HT (Applied Biosystems) real‐time PCR machine for 40 cycles, and analyzed with automatic threshold for the different assays, using water as negative control. Duplicate samples with high Ct‐variations (SD > 0.17) were rerun together with all of that patient's samples, on the same plate. This minimized the variation between qRT‐PCR values for the patients’ day 1–7 samples. *GAPDH* (Hs02758991_g1, Lot:1449046) was used as a reference gene. The mRNA expressions of target genes in relation to *GAPDH* were expressed as ratios calculated by the ΔΔCt method [2^–([CtTarget‐CtNegC]–[CtGAPDH–CtNegC])^]. As the negative control (Ct = 40) always turned out negative, the method could be described as [2^–([CtTarget]–[CtGAPDH])^].

The individual ratios had a mean interassay variation of 23% for all measurements.

### 2.4 DNA microarray‐based genotyping

*S. aureus* isolates were characterized using the StaphyType DNA microarray kit (Alere Technologies GmbH, Jena, Germany). This array simultaneously detects 333 target sequences, including species markers, resistance‐ and virulence‐associated genes, as well as typing markers allowing isolates to be assigned to MLST clonal complexes (CCs). Protocols and procedures as well as primer and probe sequences have been previously described in detail.^27^ In brief, *S. aureus* isolates were stored at −40°C or −80°C in commercially available cryotubes (various brands), then grown on Columbia blood agar and incubated overnight at 37°C. Staphylococcal cells were enzymatically lysed prior to DNA preparation using enzymes from the StaphyType DNA microarray kit, Proteinase K, and commercially available spin columns (Qiagen, Hilden, Germany). The resulting purified DNA was used as template in a linear primer elongation with one primer per target. All targets were amplified simultaneously, leading to incorporation of biotin‐16‐dUTP into the single‐stranded amplicons. Amplicons were stringently hybridized to the microarray, followed by washing steps. A horseradish‐peroxidase–streptavidin conjugate was added, and after further incubation and washing, hybridizations were visualized by a local dye precipitation. An image of the microarray was taken and analyzed using a designated reader, software, and database. This procedure allowed the detection of individual target genes as well as assignment of isolates to CCs by an automated comparison of hybridization profiles with a reference database.

### 2.5 Statistical analysis

Descriptive statistics are presented as median and interquartile range (25th, 50th, and 75th percentiles) together with minimum and maximum values. For comparison between groups, the non‐parametric Mann–Whitney test (comparison between two groups) or the Kruskal–Wallis test (comparison between more than two groups) was used. The Spearman rho test was used to assess correlations between two variables. The nonparametric Friedman test was used to evaluate differences over time. Differences were considered statistically significant at *p* < .05. All statistical analyses were performed with SPSS version 22 (IBM Corp., Armonk, NY).

### 2.6 Ethics statement

The study was conducted in accordance with the ethical guidelines of the Declaration of Helsinki, and was approved by the Regional Ethical Review Board of Uppsala, Sweden (ref: 2012/018). Written informed consent to participate was provided by all patients within the SAB cohort. For the controls, the study did not require ethical approval according to paragraph 4 of the Swedish Act concerning Ethical Conduct in Human Research (2003:460) as the blood was withdrawn at the time of blood donation (no extra harm or risk to the donors), and no personal data were collected.

## 3 RESULTS

### 3.1 Patient characteristics

Clinical characteristics of the 20 patients with SAB included in this study are summarized in Table 1. Patients were evaluated according to disease severity; 13 patients (65%) fulfilled the criteria for sepsis, and 14 patients (70%) had complicated SAB. Comorbidities according to Charlson score were recorded and 15 patients (75%) had one or more comorbidities. Ten patients had indwelling devices, of which five had metastatic foci adjacent to the indwelling material. Echocardiography was performed in 15 patients, including all patients with intracardiac devices. One patient died on day 3 after enrolment, and was therefore subject to sampling only on assessment days 1 and 2.

**Table 1:** Clinical characteristics of the study patients

Patient characteristics	(*N* = 20) No. (%)^a^
Age, y, median (range)	79 (41–92)
Male sex	16 (80)
Sepsis and bacteremia classification	
Sepsis‐3 (acute change in SOFA score ≥2)	13 (65)
Complicated bacteremia	14 (70)
Uncomplicated bacteremia	6 (30)
Comorbidity (Charlson score)	
Congestive heart failure or a history of acute myocardial infarction	9 (45)
Chronic renal insufficiency	2 (10)
Immunosuppression	2 (10)
Diabetes mellitus	4 (20)
Chronic obstructive pulmonary disease	2 (10)
Cerebrovascular lesions	3 (15)
Dementia	1 (5)
Malignancy	1 (5)
Connective tissue disease	1 (5)
Other predisposing conditions	
Intravenous drug use	1 (5)
Alcoholism	1 (5)
Indwelling prosthesis^b^	
Cardiac valve, pacemaker, implantable cardioverter defibrillator	6 (30)
Prosthetic joint devices, other orthopedic implants	5 (25)
C‐reactive protein (CRP) level	
CRP level (mg/l) on day 1^c^, median (range)	249 (57–432)
Days of adequate antimicrobial treatment at inclusion	
0–1	11 (55)
2	8 (40)
3	1 (5)
Acquisition of bacteremia	
Health care	9 (45)
Community	11 (55)
Primary focus of infection	
Infective endocarditis	4 (20)
Osteomyelitis and/or septic arthritis	6 (30)
Deep‐seated abscesses	4 (20)
Skin and soft tissue infection	1 (5)
Catheter‐associated SAB	3 (15)
Sepsis with no identifiable focus	2 (10)

^a^ Unless indicated otherwise.

^b^ Patients may have more than one indwelling prostheses.

^c^ Data missing for two patients.

### 3.2 Antibiotic susceptibility testing

Antibiotic susceptibility testing revealed that all 20 SAB isolates were sensitive to cefoxitin, and were thus classified as methicillin‐sensitive *S. aureus*.

### 3.3 Caspase‐1 activity in neutrophils and monocytes from patients with SAB and controls

**Figure 1:**
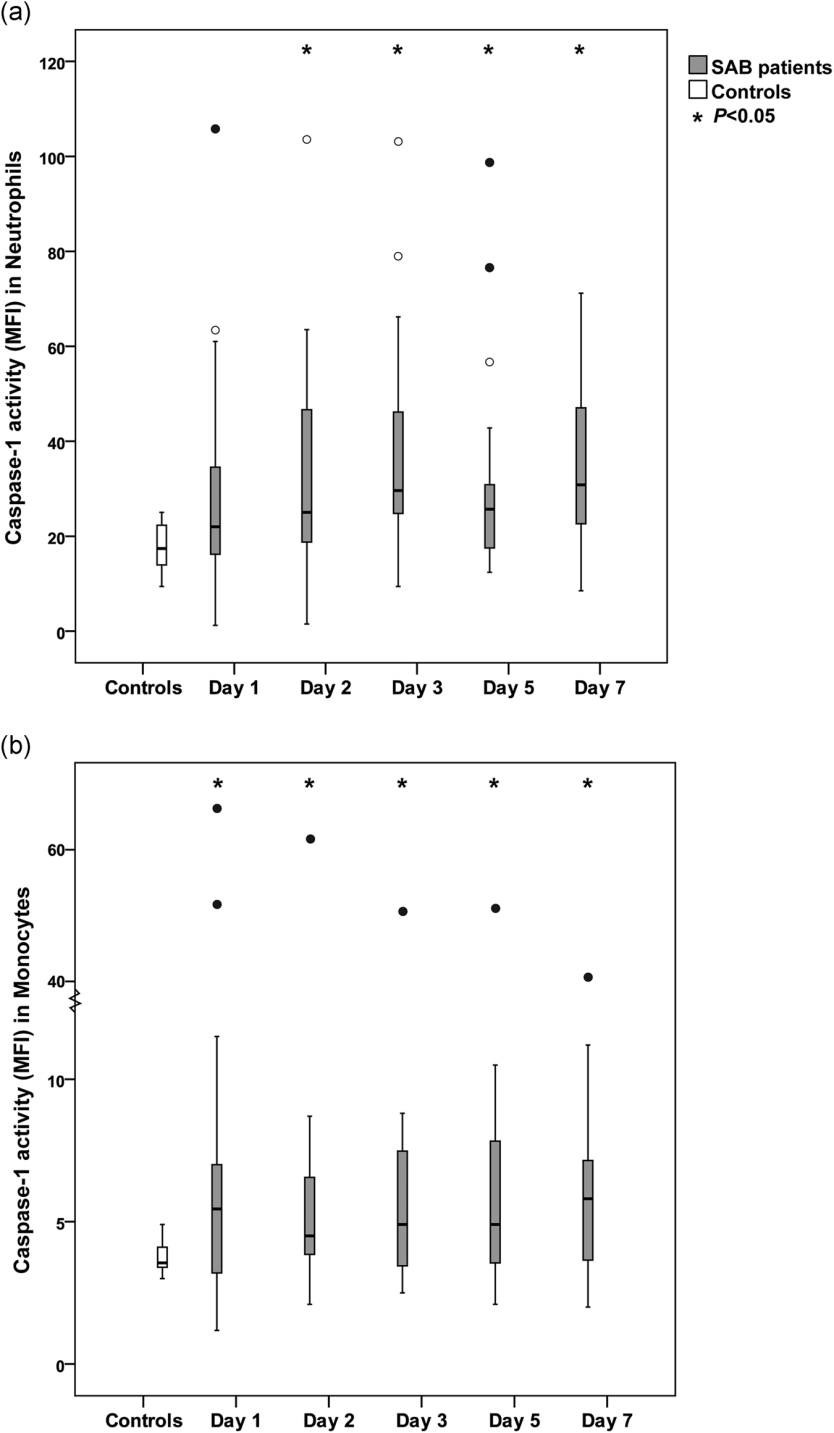
a–b Caspase‐1 activity in neutrophils and monocytes in patients with SAB and controls. Whole blood from patients with SAB (*n* = 20) was analyzed for caspase‐1 activity in (a) neutrophils and (b) monocytes by repeated measurement during the first week from SAB diagnosis and compared with healthy controls (*n* = 20). Caspase‐1 activity was analyzed by flow cytometry detecting FLICA. Values are presented as medians and interquartile ranges. The nonparametric Mann–Whitney test was used to compare patients and controls. Asterisks (*) represent significant differences between patients with SAB and controls for each day. Circles (○) represent outliers more than 1.5 box lengths from the box, and filled circles (•) represent outliers more than 3 box lengths from the box

Flow cytometry was used to determine caspase‐1 activity in neutrophils and monocytes by repeated measurement over time during the first week after SAB diagnosis. Patients with SAB displayed higher caspase‐1 activity in both neutrophils and monocytes compared with healthy controls, with significant differences at all assessment points, except for caspase‐1 activity in neutrophils on day 1 (Figure 1). Caspase‐1 activity was higher in neutrophils than in monocytes, both in bacteremia patients and in controls. During the first week after SAB diagnosis, caspase‐1 activity (measured as median MFI) ranged between 22.0 (day 1) and 30.8 (day 7) in neutrophils (range 1.2–105.8) and between 4.5 (day 2) and 5.8 (day 7) in monocytes (range 1.2–65.1). Overall, patients with SAB demonstrated a higher interindividual variation of caspase‐1 activity in both neutrophils and monocytes compared with controls, who displayed a less dispersed level of caspase‐1 activity (Figure 1). For controls, the median MFI of the caspase‐1 activity was 17.4 (9.4–25.0) in neutrophils and 3.6 (3.0–4.9) in monocytes.

The median interval from reported onset of illness to inclusion was 4 days (range 1–9). There were no correlations between reported duration of illness and caspase‐1 activity on day 1 in either monocytes (*r* = 0.18; *p* = .32) or neutrophils (*r* = 0.24; *p* = .32).

### 3.4 Proinflammatory cytokines IL‐18 and IL‐1β in patients with SAB and controls

**Figure 2:**
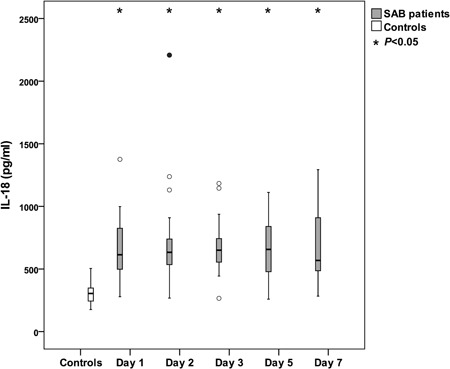
Levels of IL‐18 during SAB. Levels of IL‐18 were measured with ELISA in plasma in patients with SAB (*n* = 20) and compared with healthy controls (*n* = 26). Data are presented as medians and interquartile ranges. The Mann–Whitney test was used to compare patients and controls. Asterisks (*) represent significant differences between patients with SAB and controls for each day. Circles (○) represent outliers more than 1.5 box lengths from the box, and filled circles (•) represent outliers more than 3 box lengths from the box

Caspase‐1 activation results in the maturation of the proinflammatory cytokines IL‐18 and IL‐1β. Our finding that patients with SAB had increased caspase‐1 activity compared with controls prompted us to analyze the presence of these mature cytokines in parallel to caspase‐1 activity. IL‐18 plasma levels were significantly elevated in patients with SAB on all assessment points when compared with controls (*p* < .0001; Figure 2). In plasma from patients with SAB, the lowest median level of IL‐18 was found on day 7 (569 pg/ml; range 283–1294 pg/ml). The median plasma level of IL‐18 in the controls was 304 pg/ml (range 176–504 pg/ml).

Plasma levels of IL‐1β were low overall, with no significant differences between the patients with bacteremia (highest median level was found on day 7; 1.7 pg/ml; range 0.1–11.4 pg/ml) and the controls (median 1.6 pg/ml; range 0.8–4.2 pg/ml).

### 3.5 mRNA expression of *NLRP3*, *CASP1*, and *IL1B*, representing inflammasome priming in patients with SAB and controls

To elucidate NLRP3 inflammasome signaling on a transcription level, mRNA expressions of *NLRP3*, *CASP1*, and *IL1B* were analyzed by quantitative PCR in whole blood of patients with SAB at all assessment points, and compared with controls. The *NLRP3* mRNA ratio levels were downregulated in patients with SAB compared with controls, with significant differences at all assessment points (*p* < .001), while the *CASP1* mRNA levels did not differ between patients and controls. In contrast to *NLRP3* expression, the mRNA level of *IL1B* was higher in whole blood from patients with SAB compared with controls during the first days of bacteremia, with significant differences on days 1, 2, and 3 (*p* = .001, *p* = .004, and *p* = .021, respectively). When analyzing the data on an individual basis and over time, the mRNA expression of *CASP1* and *IL1B* coincided and followed the same pattern. Evaluating the dynamics of *CASP1* and *IL1B* expression from day 1 to day 7 revealed significant differences (*p* = .005 and *p* = .005, respectively) with decreasing levels over time, although no differences were found regarding *NLRP3* mRNA expression (*p* = .238). In addition, no differences in mRNA expression for the studied genes at any assessment point could be observed depending on normal or high level of neutrophil counts (≤8 × 10^9^/L vs >8 × 10^9^/L). Neutrophil counts on the different assessment days for all patients with SAB are provided as supporting information.

### 3.6 Inflammasome signaling in patients with complicated versus uncomplicated SAB and sepsis versus no sepsis

**Figure 3:**
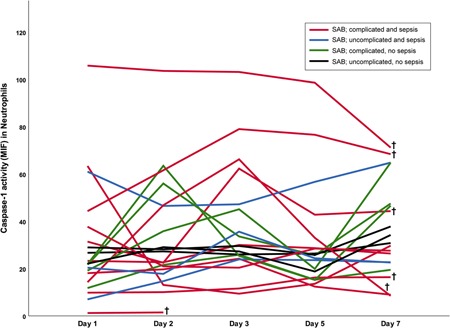
Individual dynamics of caspase‐1 activity in neutrophils for patients with SAB. Whole blood from patients with SAB (*n* = 20) was analyzed for caspase‐1 activity in neutrophils, by flow cytometry detecting FLICA. In total, 13 patients fulfilled the criteria for sepsis, and 14 patients had complicated SAB. Patients with sepsis and complicated SAB (red line; *n* = 10) are shown in relation to those with sepsis and uncomplicated SAB (blue line; *n* = 3), no sepsis but complicated SAB (green line; *n* = 4), and finally patients with no sepsis and uncomplicated SAB (black line; *n* = 3). Nonsurvivors (patients who died within 60 days according to attributable mortality definition) are indicated in figure with dagger (†) [Color figure can be viewed at wileyonlinelibrary.com]

There was no significant difference in caspase‐1 activity, nor cytokine levels of IL‐18 and IL‐1β depending on disease severity assessed as complicated (*n* = 14) versus uncomplicated (*n* = 6) SAB, or sepsis (*n* = 13) versus no sepsis (*n* = 7). However, some of the patients presenting with both sepsis and complicated bacteremia generally showed more extreme interindividual variation in caspase‐1 levels with either high or low levels, although there was an overlap with other patients with SAB (Figure 3). In addition, inflammasome signaling measured as caspase‐1 activity in neutrophils, IL‐18, or IL‐1β did not differ between SAB patients with (*n* = 15) or without comorbidities (*n* = 5) on any of the sampling days.

**Figure 4:**
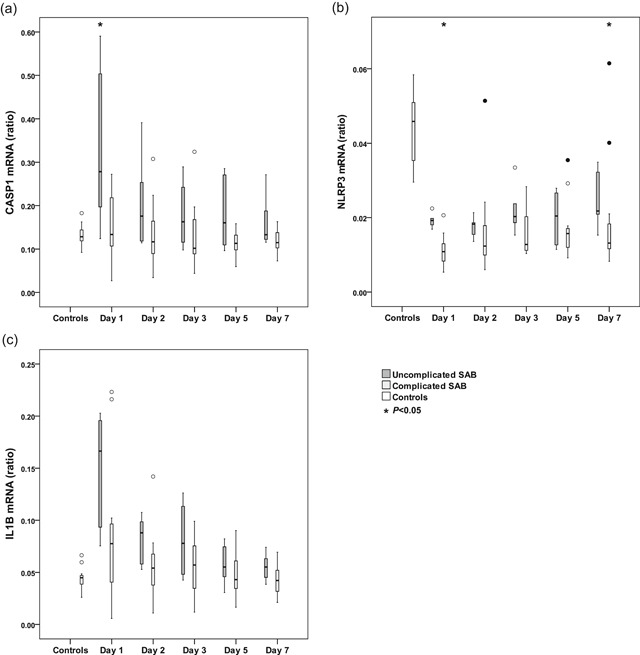
a–c. Dynamic variation in mRNA ratio of *CASP1* (*procaspase‐1*), *NLRP3*, and *IL1B* (*pro‐IL‐1β*) in patients with uncomplicated (*n* = 6) or complicated (*n* = 14) SAB and in controls (*n* = 20). The mRNA levels of (a) *CASP1*, (b) *NLRP3*, and (c) *IL1B* were measured by qPCR on cDNA from mRNA, isolated from whole blood. Ratios were calculated in relation to the reference gene *GAPDH*. Data are presented as medians and interquartile ranges. Differences in mRNA ratios between patients with complicated and uncomplicated SAB were compared with the Mann–Whitney test, and asterisks (*) represent significant differences between those SAB groups. Circles (○) represent outliers more than 1.5 box lengths from the box, and filled circles (•) represent outliers more than 3 box lengths from the box. cDNA, complementary DNA; mRNA, messenger RNA; qPCR, quantitative PCR

When evaluating mRNA levels, patients with complicated SAB generally expressed lower mRNA levels of *NLRP3*, *CASP1*, and *IL1B* compared with patients with uncomplicated SAB as shown in Figure 4a–c. However, significant differences between complicated and uncomplicated SAB were found only for mRNA *NLRP3* ratio on day 1 (*p* = .001) and on day 7 (*p* = .046), and for *CASP1* ratio on day 1 (*p* = .020). Yet, there was no difference in mRNA levels when comparing SAB patients with and without sepsis.

### 3.7 Inflammasome signaling in survivors and nonsurvivors

**Figure 5:**
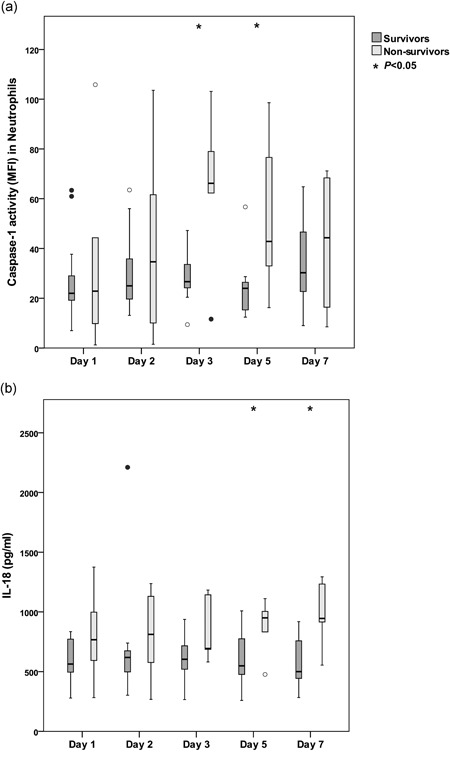
a–b. Caspase‐1 activity and levels of IL‐18 in plasma during SAB in survivors and nonsurvivors. Whole blood from patients with SAB (*n* = 20) was analyzed for (a) caspase‐1 activity in neutrophils by flow cytometry detecting FLICA and (b) plasma levels of IL‐18 were measured with ELISA. Data are presented as medians and interquartile ranges. Survivors (*n* = 14) were compared with nonsurvivors (*n* = 6) using the Mann–Whitney test, and asterisks (*) represent significant differences between groups for each day. Circles (○) represent outliers more than 1.5 box lengths from the box, and filled circles (•) represent outliers more than 3 box lengths from the box

Six patients died within 60 days according to attributable mortality definition; their median time to death was 16 days (range 3–60 days). Median age was 84 years for nonsurvivors and 74 years for survivors. Overall, nonsurvivors showed higher caspase‐1 activity in neutrophils compared with survivors, with statistically significant differences between the groups on day 3 (*p* = .044) and day 5 (*p* = .034; Figure 5). Analyzing the individual caspase‐1 levels in neutrophils for each SAB patient, nonsurvivors mainly showed either high or low levels, and all six patients who died fulfilled the criteria for both sepsis and complicated bacteremia (Figure 3). No difference in caspase‐1 activity was detected in monocytes of survivors and nonsurvivors. One patient, who died on day 3, demonstrated undetectable caspase‐1 activity in monocytes and very low caspase‐1 activity in neutrophils on day 1 and day 2.

As for caspase‐1, IL‐18 secretion was more pronounced in nonsurvivors compared with survivors, with a significant difference found on day 7 (*p* = .007; Figure 5). Levels of IL‐1β did not differ between survivors and nonsurvivors at any time point. In addition, survivors and nonsurvivors did not differ in terms of mRNA expression of *NLRP3*, *CASP1*, and *IL1B*, with the exception of *NLRP3* expression on day 5 (*p* = .014).

### 3.8 DNA microarray analysis

DNA microarray analysis demonstrated assignment of SAB isolates to nine different CCs, of which CC45 (*n* = 5), CC15 (*n* = 4), and CC1 (*n* = 3) were the most prevalent. The remaining CCs (CC6, 7, 12, 25, 30, and 97) comprised one to two isolates each.

The isolates were distributed between *agr* groups I, II, and III. Ten SAB isolates harbored *agr* group I alleles, while *agr* group II and *agr* group III alleles were found in five isolates each. The isolates carried surface‐associated capsular polysaccharide genes associated with either serotype 5 (*n* = 3) or serotype 8 (*n* = 17). Of the *S. aureus* pore‐forming toxins known to activate the NLRP3 inflammasome, the *hla* gene encoding α‐hemolysin and the *hlgA* gene encoding γ‐hemolysin were found in all isolates, whereas the *hlb* gene encoding β‐hemolysin was harbored by 10 isolates. The Panton–Valentine leukocidin genes (*lukF‐PV*, *lukS‐PV*) were found in only one isolate, whereas the leukocidin genes *lukA* and *lukB* were present in all isolates.

There were no significant differences in caspase‐1 activity in either monocytes or neutrophils depending on clonality, agr group assignment, capsular polysaccharide type, or prevalence of the β‐hemolysin gene.

## 4 DISCUSSION

Previous studies focusing on *S. aureus*‐mediated NLRP3 inflammasome activation have mainly been performed *in vitro* or in murine models.^6^, ^7^, ^16^, ^17^, ^18^, ^19^ This study investigated the activation of the caspase‐1 inflammasome during the acute phase of infection, by repeated measurement over time, in a cohort of patients with SAB, all with methicillin‐sensitive *S. aureus*. We found, in comparison to healthy controls, that patients with SAB generally displayed higher caspase‐1 activity in both neutrophils and monocytes during the first week of bacteremia, as well as elevated plasma levels of the proinflammatory cytokine IL‐18. In addition, the mRNA expression of *NLRP3*, *CASP1*, and *IL1B* was altered in patients with SAB compared with healthy controls, indicating a modified priming state of the NLRP3 inflammasome during bacteremia.

Caspase‐1 activity was measured by flow cytometry in both neutrophils and monocytes, and a clear difference was observed in caspase‐1 activity between the two leukocyte populations. Overall, caspase‐1 activity was higher in neutrophils than in monocytes, in line with previous results from our group.^8^ So far, studies have mainly focused on the NLRP3 inflammasome signaling in monocytes and macrophages,^6^, ^7^ whereas the immune response involving the NLRP3–caspase‐1–IL‐1β axis in neutrophils is less studied. In humans, neutrophils maintained in the circulation can rapidly be recruited to the site of infection and thereby play a highly important protective role in the immune response against *S. aureus* infections. In addition, expression of the NLRP3 protein and other inflammasome components has been demonstrated in neutrophils,^28^ and neutrophil‐mediated IL‐1β has been suggested to play a major role in the innate immune response.^29^ Increased caspase‐1 activity in neutrophils has also been demonstrated during *Helicobacter pylori* infection.^9^ These studies, taken together with our data showing a significant caspase‐1 activity in neutrophils, highlight the importance of neutrophils in NLR signaling during infections such as SAB.

In accordance with the finding of an increased caspase‐1 activity, plasma levels of the caspase‐1‐mediated cytokine IL‐18 were elevated in patients with bacteremia compared with controls, as previously reported in neonates with sepsis.^30^ However, in contrast to IL‐18, levels of IL‐1β were low in both patients with bacteremia and controls. It is possible that an initial release of IL‐1β had occurred already before collection of the first study sample, as IL‐1β is known to be present during the early phase of bacteremia or sepsis.

Considering the inflammasome activation on an individual basis, most but not every one of the patients with bacteremia showed increased caspase‐1 activity compared with controls. Furthermore, there was a large interindividual difference in both caspase‐1 activity and plasma levels of IL‐18 among patients with bacteremia. These results are in agreement with our previous findings demonstrating interindividual variations in caspase‐1 activity upon stimulation *ex vivo* of *Propionibacterium acnes*.^8^ Host factors as well as pathogen virulence factors may have an impact on innate immune signaling, and in addition, the inflammasome activation may be tissue specific.^31^ In patients presenting with complicated SAB or sepsis, the variation in caspase‐1 activity was more pronounced, indicating the importance of a balanced activation of the inflammasome for disease manifestation.

In our limited cohort of patients with SAB, the attributable mortality was 30%. Plasma levels of IL‐18 were higher in nonsurvivors than in survivors at all assessment points, with significant differences on day 7. These results are in agreement with previous studies pointing out IL‐18 as an important predictor of mortality in humans with severe sepsis and septic shock,^32^ as well as an IL‐18‐dependent increase in mortality in neonates with sepsis.^30^ Moreover, we observed a propensity for increased caspase‐1 activity among nonsurvivors compared with survivors. Kebaier *et al*.^16^ found NLRP3‐deficient mice to have less severe *S. aureus* pneumonia, which could indicate a potential therapeutic role of NLRP3 inhibition as an adjuvant treatment in *S. aureus* infections.^16^ By contrast, one patient in our study who died on assessment day 3 displayed undetectable caspase‐1 activity in monocytes on the day prior to death. These data suggest that patients who are either high or extremely low responders in NLRP3 inflammasome signaling might be more vulnerable to *S. aureus* disease progression and death. In recent years, a host response of simultaneous pro‐ and anti‐inflammatory nature has been recognized to prevail during sepsis, and a balanced response is now considered to produce a more favorable disease outcome.^23^ It is of interest to note that caspase‐1 is also responsible for the maturation of the anti‐inflammatory cytokine IL‐37, which binds to IL‐18R.^33^ Future studies should determine the levels of this cytokine in parallel to, for example, IL‐1β and IL‐18. Such a panel of pro‐ and anti‐inflammatory cytokines would describe the immune status of each patient at a certain time point during the course of bacteremia and sepsis.

Various *S. aureus* clones display different combinations of virulence genes encoding important virulence factors, such as exotoxins.^15^ Pore‐forming toxins known to activate the NLRP3 inflammasome include hemolysins and leukotoxins.^6^, ^7^, ^16^, ^17^, ^18^, ^19^ In particular, α‐hemolysin has been found to cause inflammasome assembling with subsequent release of IL‐1β.^6^, ^16^, ^17^ The leukotoxin PVL and the more recently discovered leukocidin A/B have also been shown to activate caspase‐1.^18^, ^19^ Our data using DNA microarray analysis showed that all SAB isolates carried the *hla*‐gene encoding α‐hemolysin and the *hlgA*‐gene encoding γ‐hemolysin. These genes are also known to be within the core genome of *S. aureus*. The genes encoding PVL were harbored by only one bacteremia isolate and did not elicit a higher caspase‐1 activity compared with others. Bacterial load and pathogen virulence are factors that may influence the magnitude of a host response.^34^ Still, we did not find caspase‐1 activity to be associated with *S. aureus* clonal lineages, of which the predominant CCs in this study are known to be among the more prevalent lineages of *S. aureus* in other Western European settings.^35^

Part of the inflammasome activity is its priming, including transcriptional upregulation of *NLRP3* and *IL1B* (the gene encoding pro‐IL‐1β).^10^ We found that SAB patients displayed an altered priming in comparison to healthy controls, with significantly lower mRNA levels of *NLRP3* accompanied by elevated levels of *IL1B* and *CASP1* (the gene encoding procaspase‐1). The individual patients’ responses to SAB were, however, highly varying in all three gene expressions. Interestingly, each patient displayed mRNA expressions of *IL1B* and *CASP1* that coincided over time, and even if the peaks in expression did not always take place during the first assessment days, their presence declined over time in all patients. While previous studies have found the NF‐κB‐mediated priming signal to trigger transcription of *NLRP3* and *IL1B*, a parallel pattern of *CASP1* and *IL1B* has not previously been reported.

When analyzing patients regarding disease severity, patients with uncomplicated SAB generally had higher *CASP1* mRNA and *IL1B* mRNA levels than patients with complicated SAB, and this pattern was pronounced on day 1. This may indicate the benefit of an adequate inflammatory response early in the bacteremia phase. By contrast, the low levels of *NLRP3* mRNA, particularly in patients with complicated SAB, suggest an immunosuppressed state, where the low expression level of *NLRP3* becomes the limiting step that prevents the assembling of the inflammasome and thereby its maturation of IL‐1β, even with *IL1B* mRNA present at an elevated level. Such an immunosuppressed state could explain the more complicated disease course of these patients. A suppressed immune response involving downregulated mRNA expression of *NLRP1* accompanied by normal levels of *IL1B* and *CASP1* mRNAs has been observed in *ex vivo* stimulated monocytes in patients with trauma,^36^ and reduced *NLRP3* gene expression in patients suffering polytrauma has also been discussed.^37^ In addition, the patients with complicated SAB in the present study displayed downregulated mRNA levels of the immunosuppression marker human leucocyte antigen‐DRA, *HLA‐DRA*, recently shown by our group,^38^ indicating an immunocompromised state.^23^

We used *GAPDH* as a reference gene, and found that it worked well in the healthy controls. However, *GAPDH* mRNA levels were not completely stable among the patients; instead, there were interindividual variations in the Ct value. This is a phenomena recently noted in patients with sepsis,^39^ and brings into question the suitability of *GAPDH* as a reference gene when analyzing mRNA in cells from individuals with severe inflammation. These findings indicate a different immunological response *in vivo* compared with *in vitro*, and suggest the regulation of the immune response during severe inflammation to be far more complex than seen in the *in vitro* models studied previously. In addition, the *GAPDH* mRNA levels were generally more increased in patients compared with controls. If taken this into calculation, the expression of *NLRP3* (shown in Figure 4b) will in fact turn out to be even more suppressed in patients with SAB. As GAPDH is a regulatory component of glucose metabolism, our finding of an unstable *GAPDH* is of greatest interest in regard to metabolic regulation of the immune system, known as immunometabolism, a phenomenon suggested to regulate immunoparalysis.^40^ Blocking GAPDH has recently been found to induce inflammasome formation, caspase‐1 activation, and IL‐1β secretion in an NLRP3‐dependent manner.^41^ Our data support this reversed interrelationship between *GAPDH* and *NLRP3*, by showing opposite expression on the transcriptional level. However, it is still not known whether, and in what way, the interrelationship between *GAPDH* and *NLRP3* contributes to protection against pathogens or plays an important role in sepsis.

Besides the use of *GAPDH* as a reference gene, this study has other limitations. Most importantly, the relatively small sample size means that it might be difficult to draw conclusions regarding host innate immune response in general, considering the heterogeneous disease manifestation of SAB. Furthermore, the cleavage process of IL‐1β/IL‐18 is complex, including other routes of activation than caspase‐1, such as enzymatic activity of serine proteases,^42^ which was not taken into consideration in this study.

In conclusion, our study shows that caspase‐1 activity was induced in innate immune cells, with subsequent release of IL‐18, in patients during the acute phase of SAB, indicating activation of the inflammasome. The large interindividual variations in caspase‐1 levels among patients with SAB suggest an important role of this part of the innate immune system for disease manifestation and outcome during bacteremia. It is tempting to suggest that individuals with the ability to activate the NLRP3 inflammasome signaling cascade in a balanced way, neither too strongly nor too weakly, may have the most favorable outcome. The mechanisms responsible for the substantial interindividual variations in host immune response deserve further evaluation. A better understanding of the individual host immune response in SAB could help tailor management and treatment of each individual patient.

## CONFLICT OF INTEREST

Stefan Monecke is an employee at Alere Technologies GmbH, Jena, Germany. However, Alere Technologies GmbH was not a commercial funder of this research. Further, they had no role in study design, data collection and analysis, decision to publish, or preparation of the manuscript. The authors have no conflict of interest to declare.

## Supporting information

Supporting information

## References

[1] Akira S , Uematsu S , Takeuchi O. Pathogen recognition and innate immunity. Cell. 2006;124:783‐801. 16497588 10.1016/j.cell.2006.02.015

[2] Kumar H , Kawai T , Akira S. Pathogen recognition by the innate immune system. Int Rev Immunol. 2011;30:16‐34. 21235323 10.3109/08830185.2010.529976

[3] Martinon F , Mayor A , Tschopp J. The inflammasomes: guardians of the body. Annu Rev Immunol. 2009;27:229‐65. 19302040 10.1146/annurev.immunol.021908.132715

[4] Kanneganti TD , Lamkanfi M , Núñez G. Intracellular NOD‐like receptors in host defense and disease. Immunity. 2007;27:549‐59. 17967410 10.1016/j.immuni.2007.10.002

[5] Sharma D , Kanneganti TD . The cell biology of inflammasomes: mechanisms of inflammasome activation and regulation. J Cell Biol. 2016;213:617‐29. 27325789 10.1083/jcb.201602089 PMC4915194

[6] Craven RR , Gao X , Allen IC , et al. Staphylococcus aureus α‐hemolysin activates the NLRP3‐inflammasome in human and mouse monocytic cells. PLoS One. 2009;4:e7446. 19826485 10.1371/journal.pone.0007446 PMC2758589

[7] Mariathasan S , Weiss DS , Newton K , et al. Cryopyrin activates the inflammasome in response to toxins and ATP. Nature. 2006;440:228‐32. 16407890 10.1038/nature04515

[8] Sahdo B , Särndahl E , Elgh F , Söderquist B. Propionibacterium acnes activates caspase‐1 in human neutrophils. APMIS. 2013;121:652‐63. 23278288 10.1111/apm.12035

[9] Pérez‐Figueroa E , Torres J , Sánchez‐Zauco N , Contreras‐Ramos A , Alvarez‐Arellano L , Maldonado‐Bernal C. Activation of NLRP3 inflammasome in human neutrophils by Helicobacter pylori infection. Innate Immun. 2016;22:103‐12. 26610398 10.1177/1753425915619475

[10] Coll R , O'Neill L , Schroder K. Questions and controversies in innate immune research: what is the physiological role of NLRP3? Cell Death Discov. 2016;2:16019. 27551512 10.1038/cddiscovery.2016.19 PMC4979470

[11] Hornung V , Bauernfeind F , Halle A , et al. Silica crystals and aluminum salts activate the NALP3 inflammasome through phagosomal destabilization. Nat Immunol. 2008;9:847‐56. 18604214 10.1038/ni.1631 PMC2834784

[12] Próchnicki T , Mangan MS , Latz E. Recent insights into the molecular mechanisms of the NLRP3 inflammasome activation. F1000Research. 2016;5:1469. 10.12688/f1000research.8614.1 PMC4963208 27508077

[13] Bauernfeind FG , Horvath G , Stutz A , et al. Cutting edge: NF‐κB activating pattern recognition and cytokine receptors license NLRP3 inflammasome activation by regulating NLRP3 expression. J Immunol. 2009;183:787‐91. 19570822 10.4049/jimmunol.0901363 PMC2824855

[14] Laupland KB . Incidence of bloodstream infection: a review of population‐based studies. Clin Microbiol Infect. 2013;19:492‐500. 23398633 10.1111/1469-0691.12144

[15] Ferry T , Perpoint T , Vandenesch F , Etienne J. Virulence determinants in Staphylococcus aureus and their involvement in clinical syndromes. Curr Infect Dis Rep. 2005;7:420‐8. 16225779 10.1007/s11908-005-0043-8

[16] Kebaier C , Chamberland RR , Allen IC , et al. Staphylococcus aureus α‐hemolysin mediates virulence in a murine model of severe pneumonia through activation of the NLRP3 inflammasome. J Infect Dis. 2012;205:807‐17. 22279123 10.1093/infdis/jir846 PMC3274379

[17] Muñoz‐Planillo R , Franchi L , Miller LS , Núñez G. A critical role for hemolysins and bacterial lipoproteins in Staphylococcus aureus‐induced activation of the Nlrp3 inflammasome. J Immunol. 2009;183:3942‐8. 19717510 10.4049/jimmunol.0900729 PMC2762867

[18] Holzinger D , Gieldon L , Mysore V , et al. Staphylococcus aureus Panton‐Valentine leukocidin induces an inflammatory response in human phagocytes via the NLRP3 inflammasome. J Leukoc Biol. 2012;92:1069‐81. 22892107 10.1189/jlb.0112014 PMC3476237

[19] Melehani JH , James DBA , Dumont AL , Torres VJ , Duncan JA . Staphylococcus aureus leukocidin A/B (LukAB) kills human monocytes via host NLRP3 and ASC when Extracellular, but not intracellular. PLoS Pathog. 2015;11:e1004970. 26069969 10.1371/journal.ppat.1004970 PMC4466499

[20] Fowler VG Jr. , Olsen MK , Corey GR , et al. Clinical identifiers of complicated Staphylococcus aureus bacteremia. Arch Intern Med. 2003;163:2066‐72. 14504120 10.1001/archinte.163.17.2066

[21] Thwaites GE , Edgeworth JD , Gkrania‐Klotsas E , et al. Clinical management of Staphylococcus aureus bacteraemia. Lancet Infect Dis. 2011;11:208‐22. 21371655 10.1016/S1473-3099(10)70285-1

[22] Singer M , Deutschman CS , Seymour CW , et al. The Third International consensus definitions for sepsis and septic shock (Sepsis‐3). JAMA. 2016;315:801‐10. 26903338 10.1001/jama.2016.0287 PMC4968574

[23] Hotchkiss RS , Monneret G , Payen D. Sepsis‐induced immunosuppression: from cellular dysfunctions to immunotherapy. Nat Rev Immunol. 2013;13:862‐74. 24232462 10.1038/nri3552 PMC4077177

[24] Cinel I , Opal SM . Molecular biology of inflammation and sepsis: a primer. Crit Care Med. 2009;37:291‐304. 19050640 10.1097/CCM.0b013e31819267fb

[25] Kim JJ , Jo EK . NLRP3 inflammasome and host protection against bacterial infection. J Korean Med Sci. 2013;28:1415‐23. 24133343 10.3346/jkms.2013.28.10.1415 PMC3792593

[26] Latz E , Xiao TS , Stutz A. Activation and regulation of the inflammasomes. Nat Rev Immunol. 2013;13:397‐411. 23702978 10.1038/nri3452 PMC3807999

[27] Monecke S , Slickers P , Ehricht R. Assignment of Staphylococcus aureus isolates to clonal complexes based on microarray analysis and pattern recognition. FEMS Immunol Med Microbiol. 2008;53:237‐51. 18507678 10.1111/j.1574-695X.2008.00426.x

[28] Ekman AK , Cardell LO . The expression and function of Nod‐like receptors in neutrophils. Immunology. 2010;130:55‐63. 20002790 10.1111/j.1365-2567.2009.03212.x PMC2855793

[29] Mankan AK , Dau T , Jenne D , Hornung V. The NLRP3/ASC/caspase‐1 axis regulates IL‐1β processing in neutrophils. Eur J Immunol. 2012;42:710‐5. 22213227 10.1002/eji.201141921

[30] Wynn JL , Wilson CS , Hawiger J , et al. Targeting IL‐17A attenuates neonatal sepsis mortality induced by IL‐18. Proc Natl Acad Sci USA. 2016;113:E2627‐35. 27114524 10.1073/pnas.1515793113 PMC4868456

[31] Melehani JH , Duncan JA . Inflammasome activation can mediate tissue‐specific pathogenesis or protection in Staphylococcus aureus infection. Curr Top Microbiol Immunol. 2016;397:257‐82. 27460814 10.1007/978-3-319-41171-2_13 PMC5145311

[32] Eidt MV , Nunes FB , Pedrazza L , et al. Biochemical and inflammatory aspects in patients with severe sepsis and septic shock: the predictive role of IL‐18 in mortality. Clin Chim Acta. 2016;453:100‐6. 26683353 10.1016/j.cca.2015.12.009

[33] Dinarello CA , Nold‐Petry C , Nold M , et al. Suppression of innate inflammation and immunity by interleukin‐37. Eur J Immunol. 2016;46:1067‐81. 27060871 10.1002/eji.201545828 PMC5003108

[34] Wang J , Roderiquez G , Norcross MA . Control of adaptive immune responses by Staphylococcus aureus through IL‐10, PD‐L1, and TLR2. Sci Rep. 2012;2:606. 22930672 10.1038/srep00606 PMC3428601

[35] Feil EJ , Cooper JE , Grundmann H , et al. How clonal is Staphylococcus aureus? J Bacteriol. 2003;185:3307‐16. 12754228 10.1128/JB.185.11.3307-3316.2003 PMC155367

[36] Relja B , Horstmann JP , Kontradowitz K , et al. Nlrp1 inflammasome is downregulated in trauma patients. J Mol Med. 2015;93:1391‐400. 26232934 10.1007/s00109-015-1320-0

[37] Horstmann JP , Marzi I , Relja B. Adrenergic stimulation alters the expression of inflammasome components and interleukins in primary human monocytes. Exp Ther Med. 2016;11:297‐302. 26889257 10.3892/etm.2015.2850 PMC4727106

[38] Rasmussen G , Cajander S , Bäckman A , Källman J , Söderquist B , Strålin K. Expression of HLA‐DRA and CD74 mRNA in whole blood during the course of complicated and uncomplicated Staphylococcus aureus bacteremia. Microbiol Immunol. 2017;61:442‐51. 28862321 10.1111/1348-0421.12533

[39] Cummings M , Sarveswaran J , Homer‐Vanniasinkam S , Burke D , Orsi NM . Glyceraldehyde‐3‐phosphate dehydrogenase is an inappropriate housekeeping gene for normalising gene expression in sepsis. Inflammation. 2014;37:1889‐94. 24858725 10.1007/s10753-014-9920-3

[40] Cheng SC , Scicluna BP , Arts RJ , et al. Broad defects in the energy metabolism of leukocytes underlie immunoparalysis in sepsis. Nat Immunol. 2016;17:406‐13. 26950237 10.1038/ni.3398

[41] Sanman LE , Qian Y , Eisele NA , et al. Disruption of glycolytic flux is a signal for inflammasome signaling and pyroptotic cell death. eLife. 2016;5:e13663. 27011353 10.7554/eLife.13663 PMC4846378

[42] Netea MG , Simon A , Van De Veerdonk F , Kullberg BJ , Van Der Meer JW , Joosten LA . IL‐1β processing in host defense: beyond the inflammasomes. PLoS Pathog. 2010;6:e1000661. 20195505 10.1371/journal.ppat.1000661 PMC2829053

