# Supplementary material for: Caspase‐1 inflammasome activity in patients with Staphylococcus aureus bacteremia
Source: Microbiol Immunol. 2019 Oct 16;63(12):487–99. doi: 10.1111/1348-0421.12738 (PMC6916170; doi:10.1111/1348-0421.12738)
Supplement: Supplementary file 1 — Supporting information [file MIM-63-487-s001.docx]

**Table Supporting information. Neutrophil counts (*10^9^/L) on different assessment days in patients with *Staphylococcus aureus* bacteremia.**

|  | Neutrophil counts (*10^9^/L ) | | | | |
| --- | --- | --- | --- | --- | --- |
| Patient ID | Day 1 | Day 2 | Day 3 | Day 5 | Day 7 |
| 1 | 11,73 | 15,94 |  |  |  |
| 2 | 12,58 | 11,93 | 9,25 | 9,44 | 15,81 |
| 3 | 7,69 | 4,91 | 10,44 | 6,19 | 5,72 |
| 4 | 4,96 | 4,32 | 3,91 | 4,84 | 4,93 |
| 5 | 7,24 | 9,23 | 10,52 | 10,63 | 11,2 |
| 6 | 9,57 | 7,79 | 8,04 | 8,04 | 8,21 |
| 7 | 21,82 | 17,24 | 14,12 | 11,01 | 8,52 |
| 8 | 17,88 | 10,01 | 8,09 | 5,99 | 6,61 |
| 9 | 17,24 | 14,49 | 14,67 | 16,50 | 16,40 |
| 10 | 12,93 | 12,37 | 10,96 | 13,41 | 16,43 |
| 11 | 7,13 | 6,29 | 5,59 | 9,36 | 9,44 |
| 12 | 6,43 | 3,15 | 3,28 | 2,99 | 4,22 |
| 13 | 6,6 | 10,5 | 9,56 | 10,82 | 11,51 |
| 14 | 2,46 | 2,58 | 2,59 | 2,52 | 2,81 |
| 15 | 13,24 | 13,93 | 13,27 | 9,71 | 10,27 |
| 16 | 7,39 | 7,20 | 7,64 | 6,26 | 5,30 |
| 17 | 4,45 | 3,69 | 2,83 | 2,75 | 2,66 |
| 18 | 3,76 | 3,21 | 3,25 | 4,82 | 5,61 |
| 19 | 6,02 | 7,13 | 6,83 | 6,13 | 6,56 |
| 20 | 6,32 | 5,58 | 4,57 | 5,12 | 4,97 |
